# Avoiding the side effects of electric current pulse application to electroporated cells in disposable small volume cuvettes assures good cell survival

**DOI:** 10.1186/s11658-016-0030-0

**Published:** 2017-01-13

**Authors:** Maciej Grys, Zbigniew Madeja, Włodzimierz Korohoda

**Affiliations:** Department of Cell Biology, Faculty of Biochemistry, Biophysics and Biotechnology, Jagiellonian University, Gronostajowa 7, 30-387 Cracow, Poland

**Keywords:** Avoiding side effects of electric current pulses, Disposable cuvettes, Reversible electroporation, Fluorescent dyes, Cell viability, Flow through electric field, Direct current electric field, Focused electric field

## Abstract

**Background:**

The harmful side effects of electroporation to cells due to local changes in pH, the appearance of toxic electrode products, temperature increase, and the heterogeneity of the electric field acting on cells in the cuvettes used for electroporation were observed and discussed in several laboratories. If cells are subjected to weak electric fields for prolonged periods, for example in experiments on cell electrophoresis or galvanotaxis the same effects are seen. In these experiments investigators managed to reduce or eliminate the harmful side effects of electric current application.

**Methods:**

For the experiments, disposable 20 μl cuvettes with two walls made of dialysis membranes were constructed and placed in a locally focused electric field at a considerable distance from the electrodes. Cuvettes were mounted into an apparatus for horizontal electrophoresis and the cells were subjected to direct current electric field (dcEF) pulses from a commercial pulse generator of exponentially declining pulses and from a custom-made generator of double and single rectangular pulses.

**Results:**

More than 80% of the electroporated cells survived the dcEF pulses in both systems. Side effects related to electrodes were eliminated in both the flow through the dcEF and in the disposable cuvettes placed in the focused dcEFs. With a disposable cuvette system, we also confirmed the sensitization of cells to a dcEF using procaine by observing the loading of AT2 cells with calceine and using a square pulse generator, applying 50 ms single rectangular pulses.

**Conclusions:**

We suggest that the same methods of avoiding the side effects of electric current pulse application as in cell electrophoresis and galvanotaxis should also be used for electroporation. This conclusion was confirmed in our electroporation experiments performed in conditions assuring survival of over 80% of the electroporated cells. If the amplitude, duration, and shape of the dcEF pulse are known, then electroporation does not depend on the type of pulse generator. This knowledge of the characteristics of the pulse assures reproducibility of electroporation experiments using different equipment.

## Background

Cell electroporation is used in many research laboratories and clinics [1–4]. Reversible electroporation is applied to introduce into cells substances which do not normally pass through cell membranes such as fluorescent dyes, peptides, RNA, antigens and genes [5–7]. In medicine, reversible and irreversible electroporation of cells and tissues is applied for drug delivery and tumor ablation [8–18].

We previously published a description of a modification of the method for electroporation. It was based on cell suspension flowing through a localized, focused, direct current electric field (dcEF). We observed that cells are sensitized to the pulsed dcEF when preincubated with presence of cationic dyes and local cationic anesthetics (e.g., lidocaine or procaine). This method has proven useful in experiments when electroporation of a large volume of cell suspension (more than 1 ml) is required and for quantitative research concerning the efficiency of cell electroporation and cell survival [17–21]. However, often only small samples of cell suspension (less than 100 μl) and only small amounts of substances introduced into cells are available for experiments. In particular, the amounts of RNA, DNA or antibodies introduced into cells are generally very limited [22–26].

Our goal was to develop a method for the preparation of disposable, simple electroporation cuvettes which can be easily inserted into commercial apparatus for horizontal electrophoresis. The construction of cuvettes and their placement in focused dcEFs was intended to avoid the dcEF pulse application side effect that commonly occur when commercially available cuvettes are used, and thus to ensure higher levels of survival of reversibly electroporated cells.

## Methods

### Chemicals

Reagents were obtained from the following suppliers: 9-aminoacridine (9-AAA), ethidium bromide, diacetate fluorescein, Alexa Fluor 488 Phalloidin, gentamicin, calcein, Lucifer yellow, phenol red; toluidine blue, lidocaine HCl, procaine HCl, tetracaine HCl and trypsin-EDTA from Sigma; fetal bovine serum (FBS) from Gibco, Invitrogen; carboxyfluorescein from Fluka-biochemist; culture medium RPMI 1640 with L-glutamine from Lonza; NaCl and sucrose from Merck; and phosphate-buffered saline (PBS) without calcium and magnesium ions and with calcium and magnesium ions from Biomed.

### Cells

Experiments were carried out on the well-characterized AT-2 rat prostate cancer cell line. Cells were grown in 25-cm^2^ Sarstedt flasks as described previously. For some of the experiments, normal human skin fibroblasts (HSF) were used [20, 27].

Before electroporation, the cells were washed in Ca^2+^- and Mg^2+^-free PBS via centrifugation, then suspended in an electroporation solution. The electroporation solution was 9.5% sucrose and PBS with Ca^2+^ and Mg^2+^ at a ratio of 19:1, unless stated otherwise.

In the sensitization experiments, cells were incubated in an electroporation solution containing 10 mM procaine HCl for 10 min. Following incubation, the cells were centrifuged for a second time and re-suspended in the electroporation solution. The efficiencies of RE or IRE as a function of the dcEFs were determined after transfer of cells to anesthetic-free and fluorescent dye-free media.

### Cell viability examination

Electroporation was carried out in a solution containing calcein, carboxyfluorescein or Lucifer yellow. The effectiveness of the method was established by scoring the number of fluorescent cells 15–30 min after electroporation.

A fluorescent viability test using fluorescein diacetate (FDA) and ethidium bromide (EtBr) was used to determine the number of live cells [20, 28–30]. During the electroporation procedure, the electroporated cells remained in the electroporation medium with or without added substances for no more than one hour. The cells were then transferred either to PBS or to the cell culture medium for examination under a fluorescence microscope. In each experiment 1250 to 2250 cells were examined under a Jenavert epi-fluorescent microscope (Carl Zeiss Jena) to determine the effect of the dcEFs for the desired exposure time. That meant that at least 250 cells were observed to determine the position of one point in the data plot.

Green fluorescent cells were counted as living and red fluorescent cells as dead with the FDA/ethidium bromide iodide test. Cells that showed uptake of calcein, carboxyfluorescein or Lucifer yellow were counted as reversibly electroporated, while cells that were not fluorescent were counted as not reversibly electroporated.

### Cell electroporation system

**Fig. 1:**
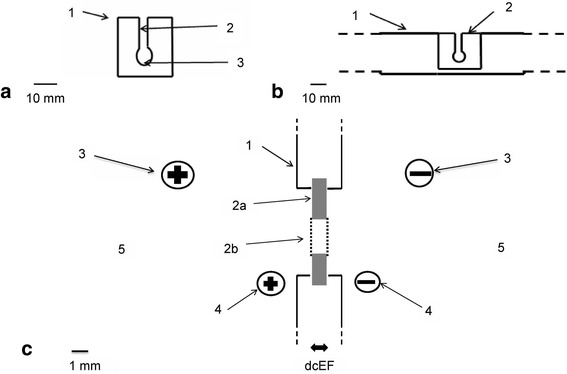
Experimental system. **a** – Electroporation cuvette made from plastic and dialysis membrane. (1) Electroporation cuvette made from plastic. (2) Canal through which cell suspension is inserted into a pocket. (3) Pocket in which the cell suspension is electroporated. Scale bar = 10 mm. **b** – Electroporation cuvette (2) inserted in the plexiglass barrier (1) in the electrophoresis apparatus. Scale bar = 10 mm. **c** – Diagram of the experimental setup applied for cell electroporation in an electroporation cuvette in a localized dcEF. (1) Transverse barrier made of plexiglass with a 19-mm diameter gap for inserting the electroporation cuvette with the cell suspension. (2) Electroporation cuvette made from plexiglass (a) and dialysis membrane (b) from Sigma dialysis tubes. (3) Power supply cords with plugs connected to the electrophoresis apparatus. (4) Probe electrodes situated at two sides of the barrier and connected to a voltmeter (Multimeter G-1004.500, RFT) to measure dcEF strength across the barrier. (5) The external solution in which the electroporation cuvette is immersed

The experiments were carried out with the system shown in Fig. 1. It consists of a standard apparatus for horizontal electrophoresis (EP 1201-1EA, Sigma-Aldrich), a dcEF pulse generator (Gene Pulser II, BioRad) or a custom-made double pulse generator, power supply cords, exchangable plexiglass cuvettes with walls made of dialysis membrane, a plexiglass barrier (3 mm thick) with an orifice for placing the electroporation cuvettes, and probe electrodes situated at two sides of the barrier and connected to a voltmeter (Multimeter G-1004.500, RTF). The disposable cuvettes inserted into the barrier had a volume of 20 μl and a depth across of 1 mm (Fig. 1).

The cell suspension was introduced into the cuvettes from above. The electroporation cuvette placed in the barrier was immersed in the same electroporation buffer in which cells were suspended.

**Fig. 2:**
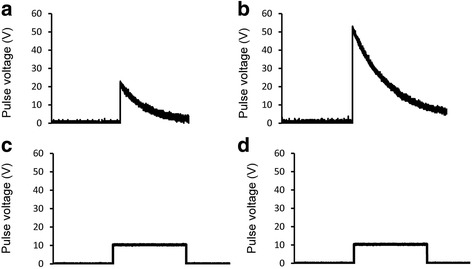
Different forms of electric pulses. Exponentially decaying electric pulse generated by Gene Pulser II. **a** – 20 V, 80 ms. **b** – 50 V, 80 ms. Square wave pulse generated by square and double pulse generator. **c** – 10 V, 80 ms. **d** – 20 V, 80 ms

The electric field was focused across the cuvette containing the cell suspension and measured in each series of experiments. Depending on the buffer solution and its volume, the dcEF across the cuvette was 50 to 60% of the voltage applied to the electrodes of the apparatus. The duration of cell suspension exposure to high dcEFs was set on the Gene Pulser II or single and double pulse generator, and the shapes of the pulses were checked with an oscilloscope (Fig. 2).

### pH measurement

The pH measurements were conducted in experimental setup consisting of a standard horizontal electrophoresis apparatus and a strip of electrophoresis paper. Phenol red was added to the tested solution at a concentration of 10 mg/100 ml as a pH change indicator. This solution contains a pH indicator which helps in monitoring pH changes in the electroporation medium during an electric field pulse. Its color exhibits a gradual transition from yellow to red over the pH range 6.8 to 8.2. Above pH 8.2, phenol red turns a bright pink (fuchsia) color.

Two electrode chambers in the apparatus were filled with different volumes of the tested solutions with phenol red and connected by a strip of paper which was earlier soaked in a given solution. The pH of each solution and the change in pH after 10 min of electric field generation were measured using a pH meter (pH-METR CP-511, Elmetron). The electric field was created between chambers by the direct current electric field generator connected to electrophoresis apparatus.

The change in pH in the electrode chambers in the electrophoresis apparatus was observed as the change in color of the phenol red. During the experiment, the current intensity was measured using a voltmeter (Multimeter G-1004.500, RFT). Photos of the solution color changes were also taken using the 13 MPx camera of a smartphone (HTC M9 Prime Camera Edition, HTC) before using the electric field (control photo), after 5 min and after 10 min.

### Statistical analysis

Statistical significance was determined using the non-parametric Mann-Whitney *U*-test with *p* < 0.01 considered to indicate significant differences.

## Results and discussion

### The principles of avoiding harmful side effects during electroporation

Electroporation methods for introducing substances that do not normally penetrate cell membranes into cells were developed in the 1970s and 80s and have since found wide application. They allow the effective introduction of proteins, DNA, RNA, fluorescent dyes and drugs into cells and tissues [1–4]. However, difficulties remain in obtaining reproducible results and preserving good cell viability during reversible electroporation. Recently published papers showed that electroporated cells die due to the many side effects associated with pulse dcEF application. Most of these harmful side effects can be avoided using a method based on the flow of cells through a locally focused electric field [11].

**Fig. 3:**
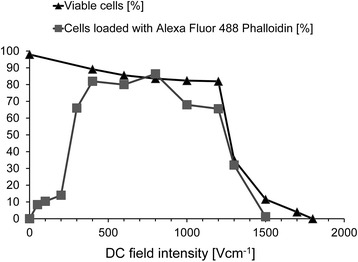
Uptake of Alexa Fluor 488 Phalloidin by AT2 cells exposed to a dcEF in a flow-through electroporation system with a localized electric field

With commercially available electroporation cuvettes, the curves for electroporation efficiency and cell viability intersect, dependent on the intensity (amplitude), duration or number of the electric dcEF pulses. The same phenomena are often observed when cell electroporation is performed on microchips [25, 31–33]. With cell flow through a focused electric field, these curves are separated and more than 80% of effectively electroporated cells survive. This is illustrated in Fig. 3.

When introducing DNA, RNA, proteins and drugs into cells, small volume electroporation cuvettes are used due to the limited availability of material and the small volume of available cell suspensions. Commercially available electroporation cuvettes typically have a volume of about 100 to 200 μl. If electrodes are in direct contact with the cell suspension, the side effects of dcEF action on cells and suspension fluids are not avoided. The experiments described here aimed to establish experimental conditions that avoid or reduce the side effects of cell electroporation when using small volume (20 μl) electroporation cuvettes.

Side effects with a significant impact on electroporated cells include:

1. Temperature changes associated with the Joule’s heat produced by the electric current flowing through the solution
2. pH changes in the solution in which the electrodes used to apply dcEF pulses are immersed in
3. Changes in electric field strength over time and in the conductivity of the solution during pulse administration, accompanied by non-uniform distribution of the electric field in the electroporation cuvette, despite the constant voltage applied to the electrodes
4. The formation of substances that are toxic for cells at the electrodes

All these phenomena occur whenever the electrodes are immersed in the electrolyte remaining in direct contact with the cells. Thus, they occur not only during electroporation or electrofusion of cells when pulses of dcEFs are applied, but also during electrophoresis of cells and proteins or cell galvanotaxis. However, they occur more slowly, thus permitting electrochemical and physical analysis. The conclusions of such studies are directly relevant to research on cell electroporation, where the same phenomena occur very quickly and in small volumes of liquids. The use of very high voltage applied to electrodes placed close to the electroporated cells and immersed in the same solution as the cells increases the severity of such side effects.

In previous research on cell electrophoresis and galvanotaxis, the side effects of electricity applied to cells have been decreased and eliminated with the following precautions:

1. The heating of the solution during the flow of electric current has been decreased by lowering the conductivity of the solution and decreasing electrolyte concentration (maintaining a constant direct current electric field) or by removing heat to cooling jackets [20, 34, 35].
  The differences in Joule’s heat production (the intensity of the electric current) does not influence the thresholds of cell response to the electric field [20, 36, 37]. The reduction in thermal effects is important in all cell electroporation experiments [38, 39]. In the experimental system for electroporation described here, besides decreasing the conductivity of the cell suspension, the electroporation cuvettes are submerged in a large volume of solution through which no current or low density current flows, and thus in which heat production is negligible [20].
2. In systems for cell electrophoresis or galvanotaxis, the harmful side effects of toxic substances produced by the electrodes (metal ions, bubbles of gas, pH changes, etc.) are eliminated by placing electrodes at a distance from the cuvettes in which the cells are suspended and focusing the dcEF in these cuvettes. The application of reversible Ag/AgCl electrodes instead of platinum, aluminum or stainless steel electrodes also reduces the formation of toxic products. Another method to eliminate the effects of electrode products on cells is to place filters (agar bridges) between the cuvettes in which cells are observed and exposed to dcEFs and the electrode chambers [36, 39–41].
3. During the application of an electric field to cells, changes in pH and conductivity occur in the solution despite the stability of the voltage applied to the electrodes. Even in systems with simple geometry such as rectangular cuvettes or a cylinder with constant cross-sections, one cannot specify the local electric field in a solution by dividing the voltage applied to the electrodes by the distance between electrodes [36, 42]. When irreversible electrodes are used (e.g., stainless steel, platinum, aluminum), the distribution of the electric field changes over time due to polarization of the electrodes. As time passes, a voltage drop begins to occur at the electrodes and reduces the electric field within the cuvette. These processes can be observed in systems where such electrodes and solutions of low conductivity are used. These phenomena also take place in systems used for cell electroporation.
  Changes resulting from the polarization of electrodes can be prevented with reversible electrodes, such as Ag/AgCl electrodes, and/or by placing the cells in a locally focused dcEF at a considerable distance from the electrodes [41]. Then the actual local electric field in the cuvette can be estimated using probe electrodes placed near the cuvette or by calculating the electric field from Ohm’s law. The local electric field in a variable geometry cuvette depends on the current intensity and the cross-sectional area through which the current flows according to Ohm’s law. When the current flowing through the system is constant (I = constants), the local strength of the electric field (V/cm) depends on the cross-sectional area through which the current flows. The smaller the cross-sectional area, the greater the local electric field at constant voltage applied to electrodes and at constant current intensity (in mA). Both methods have been studied for electrophoresis and galvanotaxis and given consistent results [20, 42].
4. Only recently have the harmful effects of local pH changes in the solution between the electrodes been investigated. It was demonstrated that in microchips these effects reduce cell survival in the vicinity of electrodes.

Łabanowski’s solution: cathode (pH = 7.2) – 15% sacharose, 3.3 mM Hepes, 6.7 mM TEA, 17.1 mM NaCl; anode (pH = 7.2) – 7% sacharose, 10 mM Hepes, 17.1 mM NaCl. Electroporation solution (pH = 7.3) – 9.5% sacharose + PBS without Ca^2+^ and Mg^2+^ in a 19:1 ratio.

There were attempts to minimize these local pH changes in the solution by increasing the concentration of buffer (i.e., the buffering capacity of the buffer solution) and determining the effect of pH on cell viability [25, 32, 33]. The pH changes in the solution under the influence of an electric current and the electrodes were tested previously in relation to electrophoresis and galvanotaxis. Increasing the concentration of the buffer and the size of the electrodes and the electrolyte concentration in the solution all reduce and slow changes in the pH of the solution in the vicinity of the electrodes, but do not eliminate them.

This problem was considered in detail in Łabanowski’s 1979 doctoral dissertation on cell electrophoresis [43]. He showed that the pH change mainly occurs due to differences in the electric charge transferring numbers of ions in various buffers and that it is not possible to eliminate changes in pH at the cathode and anode using a single buffer solution. Consequently, he developed appropriately selected buffers for the anodic and cathodic chambers to stabilize the pH near the electrodes for a few hours despite the ongoing flow of current through the solution.

**Fig. 4:**
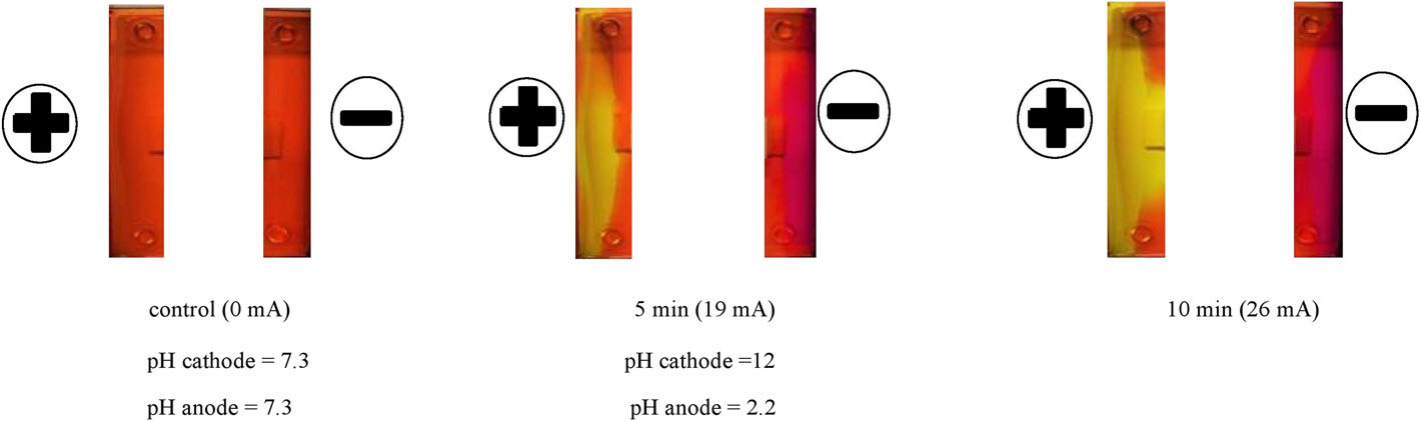
Change in the pH of a 0.9% NaCl solution after treatment with a 300 V electric current (10.9 mA)

To illustrate this, we ran appropriate experiments in the system in horizontal electrophoresis. The Sigma electrophoresis apparatus was used in our experiments. Our results (Tables 1 and 2, Fig. 4) confirmed Łabanowski’s conclusion. As observed later in experiments on cell galvanotaxis, these buffers provide stability of pH even when an electric current is applied for several hours. Moreover, the cells in the electrode buffers suggested by Łabanowski for anode and cathode solutions ensured cell survival for several hours (Table 3).

**Table 1:** Change in the pH of a 0.9% NaCl solution after 10 min treatment with a 200 V electric current according to volume solution in electrode chamber (paper strip 20 mm)

	pH at a given time			
Volume of 0.9% NaCl (ml) in the electrode chamber	Cathode		Anode	
	0 min	10 min	0 min	10 min
15	7.3	12	7.3	2.2
25	7.3	11.3	7.3	2.7
50	7.3	11.4	7.3	3
100	7.3	10.3	7.3	4

**Table 2:** Change in the pH in different solutions after treatment with a 200 V electric current (paper strip 2 mm)

	pH at a given time			
Solution	Cathode		Anode	
	0 min	10 min	0 min	10 min
Łabanowski’s solution	7.2	7.2	7.2	7.2
Electroporation solution	7.3	7.4	7.3	6.5

**Table 3:** The viability of AT2 cells estimated using the EtBr/FDA test after one hour in Łabanowski’s solution

Łabanowski’s solution	Viability of AT2 cells (%)
Control	98.8
Cathode solution	98
Anode solution	99
Cathode solution + anode solution	97.9

### Experimental verification of considerations for avoiding electroporation side effects

**Fig. 5:**
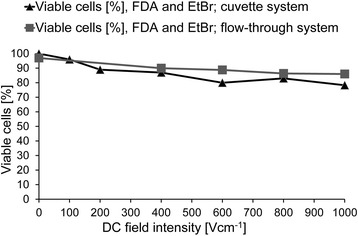
Viability of AT2 cells in a DC electric field in the flow-through system or cuvette system for 80 ms

In the first series of experiments, we examined the survival of cells suspended in disposable electroporation cuvettes, placed in locally focused electric fields, and treated with dcEF pulses of amplitude up to 1000 V cm with a pulse duration of 80 ms. These conditions provide effective electroporation of most eukaryotic cells with a diameter greater than 5 μm, shown with the method of cell suspension flow through a focused electric field in (Fig. 5). More than 90% of the electroporated cells survived such dcEF pulses in both systems thanks to the elimination of side effects related to the electrodes.

**Fig. 6:**
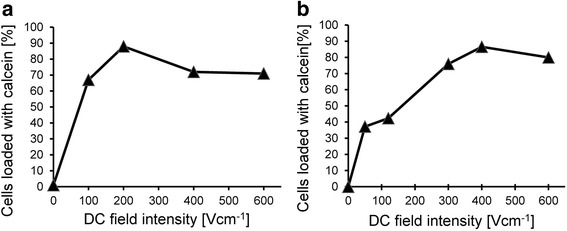
AT2 prostate cancer cell loading with calcein following a single 80 ms exposure to dcEF. **a** – Electroporation cuvette system. **b** – Flow-through electroporation system

In subsequent experiments, we compared the loading of AT2 cells with calcein via the application of 80 ms pulses of a dcEF varying in intensity in the flow through field and in the disposable cuvettes placed in a focused electric field (Fig. 6). In both cases, a pulse intensity of less than 500 V/cm caused the loading of more than 80% of cells.

**Fig. 7:**
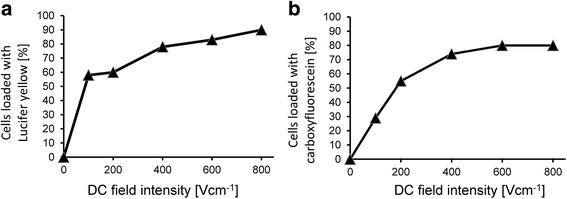
Electroporation (**a** – Lucifer yellow, **b** – carboxyfluorescein) of AT2 cells in a DC electric field for 9 ms in a solution of 6.75% sucrose + 5.3% PBS + 0.2% NaCl (1 mm) in a cuvette electroporation system

Similar results were obtained by loading AT2 cells with Lucifer yellow or carboxyfluorescein in the cuvette electroporation system (Fig. 7).

**Fig. 8:**
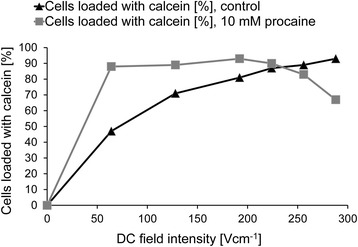
Electroporation of AT2 cells (calcein) in a DC electric field in the presence or absence of 10 mM procaine for 50 ms (square wave pulse generator) in a cuvette electroporation system

Using the disposable cuvette system, we also confirmed the sensitization of cells to a dcEF by procaine [23] by observing the loading of AT2 cells with calcein and using a square pulse generator, applying 50 ms single rectangular pulses (Fig. 8). In this case, more than 90% of cells were effectively electroporated and loaded with calcein.

## Conclusions

Direct contact of the electrodes with a small volume of liquid in which the cells are placed is harmful to cells, which are killed not only by the electric field acting on them but also indirectly, due to other side effects. Our results suggest that experiments concerning electroporation and electrofusion of cells should not be conducted in isolation from other research on the effects of electric current and electric fields on cells, in phenomena such as galvanotaxis or cell electrophoresis.

In research on cell electrophoresis and cell galvanotaxis, methods were developed for reducing harmful side effects, such as changes in the pH and heterogeneity of the electric field near the electrodes, changes in the field intensity between the electrodes, toxic electrode products and gas bubbles. As shown in this study, using previously developed methods for avoiding the side effects of electric current pulse application to cells also improves the viability of electroporated cells.

Cell death due to the side effects of electric current pulses acting on cells can be avoided during electroporation. It is possible to exclude effects related to non-physiological pH, toxic electrode products and non-uniformity of dcEF intensity fields acting on the cells by:

1. Placing the cells at a considerable distance from the electrodes and avoiding direct contact of the cell suspensions with electrodes
2. Separating liquids containing cells from electrodes with dialysis membranes or agar bridges
3. Using reversible electrodes, preferably Ag/AgCl electrodes
4. Preventing heating of cell suspensions by placing electroporation cuvettes in a water jacket and decreasing the medium conductivity and Joule’s heat production by the electric current

Under these conditions, the yield of effective reversible electroporation of cells exceeds 80%. The side effects of applying electric fields to cells should be decreased or eliminated, particularly in experiments in which individual cells are investigated.

## Abbreviations

dcEF: Direct current electric field
EtBr: Ethidium bromide
FBS: Fetal bovine serum
FDA: Fluorescein diacetate
IRE: Irreversible electroporation
PBS: Phosphate-buffered saline
RE: Reversible electroporation

## References

[1] Teissié J, Eynard N, Vernhes M, Bénichou A, Ganeva V, Galutzov B, Cabanes P (2002). Recent biotechnological developments of electropulsation. A prospective review. Bioelectrochemistry.

[2] Gehl J (2003). Electroporation: theory and methods, perspectives for drug delivery, gene therapy and research. Acta Physiol Scand.

[3] Favard C, Dean D, Rols M (2007). Electrotransfer as a non viral method of gene delivery. Curr Gene Ther.

[4] Ferraro B, Cruz Y, Coppola D, Heller R (2009). Intradermal delivery of plasmid VEGF165 by electroporation promotes wound healing. Mol Ther.

[5] Neumann E, Schaeffer-Ridder M, Wang Y, Hofschneider PH (1982). Gene transfer into mouse lymphoma cells by electroporation in high electric fields. EMBO J.

[6] Bodles-Brakhop A, Heller R, Draghia-Akli R (2009). Electroporation for the delivery of DNA-based vaccines and immunotherapeutics: current clinical developments. Mol Ther.

[7] Pakhomova O, Gregory B, Pakhomov A (2013). Facilitation of electroporative drug uptake and cell killing by electrosensitization. J Cell Mol Med.

[8] Miller L, Leor J, Rubinsky B (2005). Cancer cells ablation with irreversible electroporation. Technol Cancer Res Treat.

[9] Nuccitelli R, Pliquett U, Chen X, Ford W, James Swanson R, Beebe S, Kolb J, Schoenbach K (2006). Nanosecond pulsed electric fields cause melanomas to self-destruct. Biochem Biophys Res Commun.

[10] Nuccitelli R, Chen X, Pakhomov A, Baldwin W, Sheikh S, Pomicter J, Ren W, Osgood C, Swanson R, Kolb J, Beebe S, Schoenbach K (2009). A new pulsed electric field therapy for melanoma disrupts the tumor’s blood supply and causes complete remission without recurrence. Int J Cancer.

[11] Nuccitelli R, Tran K, Sheikh S, Athos B, Kreis M, Nuccitelli P (2010). Optimized nanosecond pulsed electric field therapy can cause murine malignant melanomas to self-destruct with a single treatment. Int J Cancer.

[12] Nuccitelli R, Berridge J, Mallon Z, Kreis M, Athos B, Nuccitelli P. Nanoelectroablation of murine tumors triggers a CD8-dependent inhibition of secondary tumor growth. Plos One. 2015;10. 10.1371/journal.pone.0134364 PMC4521782 26231031

[13] Li S (2008). Electroporation protocols. Preclinical and clinical gene medicine.

[14] Ueki T, Uemura H, Nagashima Y, Ohta S, Ishiguro H, Kubota Y (2008). Antitumor effect of electrochemotherapy with bleomycin on human prostate cancer xenograft. BJU International.

[15] Rubinsky J, Onik G, Mikus P, Rubinsky B (2008). Optimal parameters for the destruction of prostate cancer using irreversible electroporation. J Urol.

[16] Gehl J (2008). Electroporation for drug and gene delivery in the clinic: doctors go electric. Methods Mol Biol.

[17] Huang Y, Rubinsky B (2003). Flow-through micro-electroporation chip for high efficiency single-cell genetic manipulation. Sens Actuators, A.

[18] Geng T, Zhan Y, Wang H, Witting S, Cornetta K, Lu C (2010). Flow-through electroporation based on constant voltage for large-volume transfection of cells. J Control Release.

[19] Geng T, Zhan Y, Lu C (2012). Gene delivery by microfluidic flow-through electroporation based on constant DC and AC field.

[20] Korohoda W, Grys M, Madeja Z (2013). Reversible and irreversible electroporation of cell suspensions flowing through a localized DC electric field. Cell Mol Biol Lett.

[21] Grys M, Madeja Z, Korohoda W (2014). Decreasing the thresholds for electroporation by sensitizing cells with local cationic anesthetics and substances that decrease the surface negative electric charge. Cell Mol Biol Lett.

[22] Fox M, Esveld D, Valero A, Luttge R, Mastwijk H, Bartels P, van den Berg A, Boom R (2006). Electroporation of cells in microfluidic devices: a review. Anal Bioanal Chem.

[23] Kim J, Hwang I, Britain D, Chung T, Sun Y, Kim D (2011). Microfluidic approaches for gene delivery and gene therapy. Lab Chip.

[24] Ziv R, Steinhardt Y, Pelled G, Gazit D, Rubinsky B (2008). Micro-electroporation of mesenchymal stem cells with alternating electrical current pulses. Biomed Microdevices.

[25] Wu M, Zhao D, Wei Z, Zhong W, Yan H, Wang X, Liang Z, Li Z (2013). Method for electric parametric characterization and optimization of electroporation on a chip. Anal Chem.

[26] Wang S, Lee L (2013). Micro-/nanofluidics based cell electroporation. Biomicrofluidics.

[27] Krzysiek-Mączka G, Korohoda W (2008). Surface anisotropy orients cell divisions in contact guided cells. Folia Biol.

[28] Szydłowska H, Zaporowska E, Kuszlik-Jochym K, Korohoda W, Branny J (1977). Membranolytic activity of detergents as studied with cell viability tests. Folia Histochem Ceytochem.

[29] Kemp RB, Meredith RW, Gamble S, Frost M (1982). A rapid cell culture technique for assessing the toxicity of detergent-based products in vitro as a possible screen for eye irritancy in vivo. Cytobios.

[30] Zaporowska-Siwiak E, Michalik M, Kajstura J, Korohoda W (1985). Density-dependent survival of Ehrlich ascites tumour cells in the presence of various substrates for energy metabolism. J Cell Sci.

[31] Yumura S, Matsuzaki R, Kitanishi-Yumura T (1995). Introduction of macromolecules into living Dictyostelium cells by electroporation. Cell Struct Funct.

[32] Li Y, Wu M, Zhao D, Wei Z, Zhong W, Wang X, Liang Z, Li Z (2015). Electroporation on microchips: the harmful effects of pH changes and scaling down. Sci Rep.

[33] Rubinsky L, Guenther E, Mikus P, Stehling M, Rubinsky B (2015). Electrolytic effects during tissue ablation by electroporation. Technol Cancer Res Treat.

[34] Kolin A (1964). Kinematic stabilization of continuous-flow electrophoresis against thermal convection. Proc Natl Acad Sci.

[35] Bangham A, Heard D, Flemans R, Seaman G (1958). An apparatus for microelectrophoresis of small particles. Nature.

[36] Korohoda W, Kurowska A (1970). Quantitative estimations of the thresholds of electrotactic responses in Amoeba proteus. Acta Protozool.

[37] Pucihar G, Kotnik T, Kandušer M, Miklavčič D (2001). The influence of medium conductivity on electropermeabilization and survival of cells in vitro. Bioelectrochemistry.

[38] Davalos RV, Rubinsky B (2008). Temperature considerations during irreversible electroporation. Int J Heat Mass Transfer.

[39] Shafiee H, Garcia P, Davalos R (2009). A preliminary study to delineate irreversible electroporation from thermal damage using the Arrhenius equation. J Biomech Eng.

[40] Abramson HA, Moyer LS, Gorin MH (1942). Electrophoresis of Proteins and the Chemistry of the Cell Surfaces.

[41] Djamgoz MBA, Mycielska M, Madeja Z, Fraser SP, Korohoda W (2001). Directional movement of rat prostate cancer cells in direct-current electric field: involvement of voltage-gated Na + channel activity. J Cell Sci.

[42] Korohoda W, Mycielska M, Janda E, Madeja Z (2000). Immediate and long‐term galvanotactic responses of Amoeba proteus to dc electric fields. Cell Motil Cytoskeleton.

[43] Labanowski J (1979). Analysis of conditions for preparative electrophoresis of cells and subcellular fractions.

